# Aflatoxin B1, Gut Microbiota Dysbiosis, and the Intestinal Barrier: Implications for the Gut–Liver Axis and Extrahepatic Cancer Risk

**DOI:** 10.3390/toxins18090361

**Published:** 2026-08-24

**Authors:** Charbel Sleilaty, Marilyn Hnein, Teddy Lattouf, Thea Gemayel, Fouad Attieh, Tia Kreidy, Kevin Sarkis, May Bark, Maha Hoteit, Alain Chebly, Marwan Ghosn, André El Khoury, Jad Chémali

**Affiliations:** 1 Faculty of Medicine, Saint Joseph University of Beirut, Beirut P.O. Box 17-5208, Lebanon; charbel.sleilaty@net.usj.edu.lb (C.S.); marilyn.hnein@net.usj.edu.lb (M.H.); teddylattouf@gmail.com (T.L.); theagem112@gmail.com (T.G.); fouad.attieh@net.usj.edu.lb (F.A.); 2 Laboratory of Cancerology and Carcinogenic Agents, Faculty of Medicine, Saint Joseph University of Beirut, Beirut P.O. Box 17-5208, Lebanon; tia.kreidy@net.usj.edu.lb (T.K.); kevin.sarkis@net.usj.edu.lb (K.S.); may.bark@usj.edu.lb (M.B.); 3 PHENOL Research Program, Faculty of Public Health, Section 1, Lebanese University, Beirut P.O. Box 6573, Lebanon; m.hoteit@ul.edu.lb; 4 Department of Primary Care and Population Health, University of Nicosia Medical School, Nicosia 2417, Cyprus; 5 Institut National de Santé Publique, d’Épidémiologie Clinique et de Toxicologie-Liban (INSPECT-LB), Beirut 1103, Lebanon; 6 Center Jacques Loiselet for Medical Genetics and Genomics (CGGM), Faculty of Medicine, Saint Joseph University, Beirut P.O. Box 17-5208, Lebanon; alain.chebly@usj.edu.lb; 7 Hematology-Oncology Department, Faculty of Medicine, Saint Joseph University of Beirut, Beirut 166830, Lebanon; marwan.ghosn@usj.edu.lb; 8 Center for Analysis and Research, Research Unit on Agro-Food Technologies and Valorization, Faculty of Sciences, Saint Joseph University of Beirut, Mar Roukos, Dekwaneh, Beirut P.O. Box 1514, Lebanon

**Keywords:** Aflatoxin B1 (AFB1), gut microbiota, intestinal barrier, gut–liver axis, mucus dysbiosis, bacterial translocation, short-chain fatty acids, chronic inflammation, hepatocellular carcinoma, carcinogenesis

## Abstract

Aflatoxin B1 (AFB1) is a potent foodborne mycotoxin classified as a Group 1 human carcinogen by the International Agency for Research on Cancer (IARC) and is widely recognized for its causal role in hepatocellular carcinoma (HCC). Beyond its well-established hepatotoxicity, increasing evidence indicates that AFB1 also disrupts intestinal homeostasis by impairing epithelial barrier integrity, reducing mucus production, altering gut microbial composition, and disturbing microbial metabolism. These changes promote increased intestinal permeability, facilitating the translocation of bacteria and microbial products and contributing to chronic inflammation through the gut–liver axis. Recent experimental studies further suggest that alterations in short-chain fatty acid (SCFA) production and microbiota-dependent signaling pathways may actively mediate AFB1-induced intestinal and hepatic injury. Although direct evidence linking AFB1-induced dysbiosis to extrahepatic carcinogenesis remains limited, growing evidence indicates that persistent barrier dysfunction, microbial imbalance, and chronic inflammation may create a microenvironment favorable for tumor initiation and progression in tissues beyond the liver. This review critically summarizes current evidence regarding the effects of AFB1 on intestinal barrier function, gut microbiota dysbiosis, bacterial translocation, microbial metabolites, and the gut–liver axis while evaluating the mechanistic evidence supporting these interactions and highlighting the major knowledge gaps that should be addressed in future research.

## 1. Background

Aflatoxins (AFs) are among the most widely distributed and hazardous mycotoxins, with an estimated 4.5 billion people subject to chronic exposure [1,2]. Around 20 distinct AF compounds have been described to date, including B1, B2, G1, G2, M1, M2, aflatoxicol, and aflatoxin Q1. Among these, AFB1 is the most extensively studied, owing to its high carcinogenic potency and its broad occurrence across agricultural commodities [2,3].

AFs are particularly common in warm and humid regions. Food contamination may occur before or after harvest and is influenced by temperature, humidity, soil characteristics, and storage conditions [4]. Climate change may further increase AFB1 contamination, particularly in maize-producing areas. Battilani et al. (2016) [5] predicted that a 2 °C temperature increase could raise the risk of aflatoxin contamination from low to moderate in several European countries, including France, Italy, and Romania.

AFM1, the hydroxylated metabolite of AFB1, is mainly found in milk and dairy products from animals exposed to contaminated feed and may also occur in human breast milk. In Europe, grains and grain-based products, particularly corn, bread, and fine bakery products, are the main contributors to chronic AFB1 exposure. The highest AFB1 and total aflatoxin concentrations are generally reported in legumes, nuts, and oilseeds, whereas AFM1 exposure is mainly linked to milk, dairy products, and milk-based foods for infants and young children [6].

In particular, aflatoxin B1 (AFB1), a secondary fungal metabolite with the molecular formula C_17_H_12_O_6_, stands out due to its high toxicity and biological activity [6].

Produced by *Aspergillus flavus* and *Aspergillus parasiticus*, AFB1 is commonly found in widely consumed food products, including cereals, peanuts, and animal feed worldwide, often co-occurring with other fungal contaminants. [7,8].

AFB1 has been designated as a Group 1 carcinogen by the International Agency for Research on Cancer (IARC), highlighting its well-established role in human carcinogenesis [9]. Epidemiological data further indicate that early-life exposure may be particularly concerning; recent studies suggest that children under five years of age consuming contaminated foods, mostly peanuts, have an increased risk of exposure, which may be associated with a higher risk of liver cancer [10]. Beyond its carcinogenic effects, exposure to AFB1 has been associated with immunosuppression, organ damage, and growth impairment [11]. It is also recognized as a major risk factor for hepatocellular carcinoma (HCC), contributing to an estimated 4.6–28.2% of cases worldwide [12].

This carcinogenic effect is largely attributed to its metabolic activation in the liver. Following ingestion, AFB1 undergoes biotransformation in hepatocytes, where it is converted into reactive intermediates capable of interacting with cellular macromolecules, a process central to its toxic and carcinogenic effects [13,14]. This metabolic conversion is primarily mediated by cytochrome P450 (CYP450) enzymes, particularly CYP1A2 and CYP3A4 [15]. As a result, AFB1 is transformed into the highly reactive AFB1-8,9-epoxide, which readily forms adducts with DNA, proteins, and other cellular targets, thereby promoting hepatotoxicity and carcinogenesis. AFB1 exposure is classically associated with a characteristic TP53 mutation at codon 249 (R249S), resulting from a G > T transversion, which represents a molecular signature of aflatoxin-induced hepatocarcinogenesis. [16,17].

**Figure 1:**
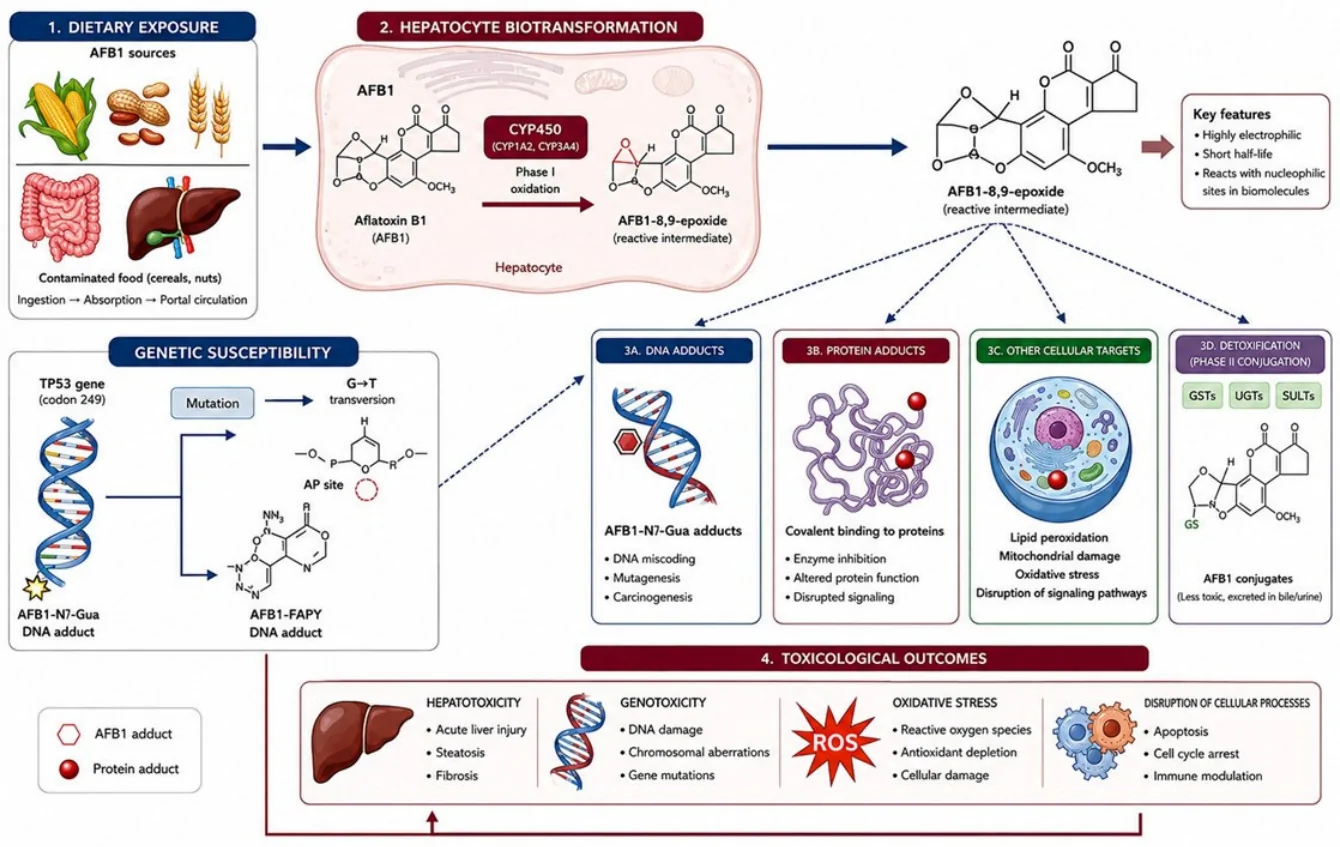
Cellular mechanisms of aflatoxin B1 (AFB1) toxicity: from metabolic biotransformation to genotoxic outcomes.

This epoxide metabolite exhibits strong reactivity toward nucleic acids, leading to oxidative stress and preferential binding to guanine residues in DNA, resulting in the formation of aflatoxin-N7-guanine adducts [18]. Such interactions can alter DNA structure, interfere with the activity of DNA-dependent RNA polymerase, and reduce RNA and protein synthesis [19]. Moreover, these genotoxic metabolites, especially AFB1-8,9-epoxide, are responsible for the formation of AFB1-DNA adducts, which represent a key step in mutagenesis and carcinogenesis [20]. In addition, this reactive compound can penetrate cellular membranes and incorporate into the genome, leading to gene mutations and disruption of protein function [21,22]. More specifically, aflatoxin B_1_ is metabolically activated by cytochrome P450 enzymes into a reactive 8,9-epoxide that binds to DNA, forming AFB_1_-N7-guanine and the more stable AFB_1_-FAPY adducts [23]. These mechanisms are summarized in Figure 1.

The interaction of AFB1 metabolites with hepatic macromolecules disrupts normal cellular processes, affecting enzymatic activity and immune responses [24,25] and further contributing to liver injury [26]. Moreover, exposure to AFB1 has been linked to epigenetic modifications, including alterations in DNA methylation, histone regulation, and non-coding RNA expression, which may influence gene expression both immediately and over time [27].

Beyond its role in the development of HCC, recent findings have demonstrated the potential impact of AFB1 on the intestinal barrier and its possible contribution to other types of cancers through mechanisms involving microbiota dysbiosis and mucus layer disruption. This review presents how high consumption of AFB1 may induce intestinal dysbiosis and alter the intestinal barrier permeability, potentially leading to bacterial translocation and contributing to carcinogenesis in extrahepatic organs.

## 2. Aflatoxin B1 and the Intestinal Barrier

AFs are primarily absorbed through the gastrointestinal tract following the ingestion of contaminated food, and the intestinal epithelium represents their first and most concentrated site of contact [28]. A clear understanding of AFB1’s impact on the intestine, particularly the small intestine, is vital, as this is the region where modifications to the intestinal microbiota and activation of immune cells are most likely to occur [29,30].

AFB1 can be absorbed in the intestine through multiple mechanisms: (a) a fraction of the toxin can cross the epithelial barrier unchanged; (b) AFB1 can enter enterocytes via passive diffusion, where it is metabolized by CYP450 enzymes into its reactive epoxide form. This metabolite binds to proteins, and the resulting complexes are subsequently transported to the liver through the portal vein. (c) Due to its lipophilicity, AFB1 may also be transported through the paracellular pathway, contributing to the disruption of tight junctions and damaging the gut epithelium [31].

This disruption is partly attributed to the reduced expression of tight junction proteins, such as zonula occludens-1 (ZO-1), which weakens intercellular junctions and increases intestinal permeability [32,33], thereby disrupting the connection between actin and the cytoskeleton [34]. This can promote increased translocation of other pathogens and toxins into the bloodstream, resulting in systemic inflammation and immune dysfunction. It also leads to malabsorption of nutrients in the small intestine and impairment of intestinal barrier function, [33,35] resulting in toxin and antigen translocation and a leak of bacteria from the gut, causing metabolic damage [36,37]. Simultaneously, multiple in vitro studies have shown the same outcomes related to the integrity of the intestinal barrier when human colon carcinoma cells (Caco-2) are exposed to different mycotoxins, including AFB1, FB1, ochratoxin A (OTA) T-2 toxin, and DON [31].

It should be noted that the in vitro evidence on AFB1 and the intestinal epithelium is not limited to Caco-2 monolayers, although Caco-2 remains the most extensively used human model. In differentiated and undifferentiated Caco-2 cells, AFB1 and its hydroxylated metabolite AFM1 both reduced cell growth, increased lactate dehydrogenase release, and caused dose- and time-dependent DNA damage, with differentiated cells (which more closely resemble mature enterocytes) being consistently more sensitive [38]. AFB1 has also been shown to reduce viability and to activate oxidative stress, mitochondrial damage, and lipid metabolism pathways in the human colorectal cell lines HCT116 and SW480 [39,40], and it decreases viability of the non-transformed human colonic epithelial cell line NCM460, with additive toxicity when combined with AFM1 [41]. The rat epithelial line IEC-6 and the human lines HT-29 and T84 have both been validated as suitable models for assessing mycotoxin-induced epithelial injury, oxidative stress, and barrier dysfunction and have been used extensively for other mycotoxins (e.g., Alternaria toxins and deoxynivalenol) [42,43], although dedicated AFB1 dose–response studies using these specific lines remain comparatively scarce and represent a priority for future in vitro work. Regarding the porcine models, most published AFB1 studies have used the jejunal line IPEC-J2 (Table 1). The related, non-immortalized line IPEC-1 has been validated for other mycotoxins such as deoxynivalenol, T-2 toxin, and fumonisin B1, where it behaves similarly to Caco-2 monolayers in terms of tight junction disruption and increased paracellular passage [44], but to our knowledge, no AFB1-specific study using IPEC-1 has yet been published; this gap is acknowledged rather than concealed and is highlighted here as a specific need for future research rather than as a basis for firm conclusions.

Furthermore, impaired intestinal epithelial cell proliferation has been also reported as a consequence of AFB1 exposure in animal models through mechanisms like G2/M cell cycle arrest in epithelial cells [45], increased apoptosis rates, and reduced goblet cells numbers [46]. PAS staining revealed that the number of goblet cells in the colon was drastically (*p* < 0.05) reduced after combined treatment [3]. Compromised cell membrane integrity leads to increased lipid peroxidation and elevated levels of malondialdehyde (MDA), a marker of oxidative stress. Several studies have reported increased MDA levels in the livers of chickens exposed to AFB1, suggesting oxidative damage and necrobiosis [45,46,47,48].

According to Tejada-Castaneda et al. (2008) [49], a 3-week trial of Ross 308 broilers chicken given 1200 ng AFB1/g of feed demonstrated that dietary AFs damaged the microvilli structure. According to the findings observed from scanning electron microscopy, microvilli were found to be shortened and fused in the duodenum, jejunum, and ileum. Moreover, tight junctions were absent, and the denatured protein appeared as irregular masses. The authors conclude that AFs cause the loss of epithelial polarity [32].

Nava-Ramírez et al. [50] reported that the AF group composed of female turkey poults fed with an AFB1-contaminated diet exhibited reduced villus height (*p* < 0.0001) and lower total villus cross-section (*p* < 0.0001). This group had the highest serum FITC-d concentration (*p* < 0.007), implying increased intestinal permeability [51]. The findings overall show that AFs promoted the proliferation of the opportunistic pathogens, resulting in *Streptococcus lutetiensis*-driven gut dysbiosis [50]. This experiment proved that AFB1 has the ability to trigger gut inflammation and increase gut permeability [32].

Wang et al. [51] demonstrated that AFB1 or high-fat diet (HFD) alone cannot provoke strong physiological changes, whereas combined AFB1 + HFD exposure significantly impaired intestinal barrier integrity, promoted hepatic lipid accumulation, and induced Th17-driven inflammation in mice [51]. The increase in cytokines was caused by exposure to both AFB1 and HFD alone, reflecting a pro-inflammatory response from cytokines like IL-6, IL-1β, TNF-α, IL-23, IL-17, IL-22, and TGF-β [51]. The combined treatment resulted in significantly increased serum MDA levels (*p* < 0.0001). Conversely, the endogenous antioxidants Glutathione (GSH) and Superoxide Dismutase (SOD) decreased significantly (both *p* < 0.0001) [51]. Changes suggest compromised antioxidant defense and increased oxidative stress [51]. In line with these results, tight junction protein ZO-1 and occludin expression was significantly decreased in the combined group (both *p* < 0.0001), indicating a marked disruption of the intestinal barrier [51]. The disruption of this barrier promotes the translocation of endotoxins, aggravating liver inflammation [51].

Notably, combined exposure resulted in synergistic effects, with higher MDA concentrations (*p* < 0.05) and lower GSH and SOD concentrations (*p* < 0.05) compared with each treatment administered separately. [51]. These results back up the proposal that concurrent exposure of AFB1 and HFD enhances oxidative stress, impairs intestinal barrier, and aggravates systemic inflammatory damage [51].

In addition, Zhang et al. [52] demonstrated that exposure to AFB1 resulted in significant changes in the morphology of the intestine. In the jejunum, villus height was significantly reduced (*p* < 0.01), whereas crypt depth was markedly increased (*p* < 0.01). Consequently, the VH/CD ratio showed a significant decrease (*p* < 0.01) [52]. It also resulted in significant changes in the integrity of the intestine. Serum DAO and D-lactate, which are markers for compromised integrity, were substantially increased (*p* < 0.01). At the same time, exposure to AFB1 also resulted in a significant decrease in the levels of both sIgA and MUC2 (*p* < 0.01) [52]. Additionally, the transcriptional and protein levels of the tight junction proteins ZO-1, occludin, and claudin-4 were also substantially lower (*p* < 0.05, *p* < 0.01) [52]. Changes in the levels of oxidative stress markers were also observed. T-AOC and GSH-Px were substantially lower, while MDA was substantially increased (*p* < 0.01) [52]. AFB1 also triggered a potent inflammatory response, with increased levels of lipopolysaccharides (LPS), IL-1β, IL-6, and TNF-α, which were significantly upregulated (*p* < 0.01) [52]. The transcription and expression of TLR4 and MyD88 were also upregulated (*p* < 0.01) by AFB1, which suggested that the TLR4/MyD88 signaling pathway was activated. It is well established that lipopolysaccharide (LPS), an inflammatory factor derived from intestinal bacteria, can bind to the TLR4/MD-2 receptor on innate immune cells [52]. This interaction activates the MyD88 signaling pathway, leading to the activation of the transcription factor NF-κB and subsequent secretion of inflammatory cytokines, ultimately promoting intestinal inflammation, as confirmed by previous studies [52].

Collectively, these findings demonstrate that AFB1 disrupts intestinal morphology, impairs mucosal protein secretion, compromises tight junction integrity, weakens antioxidant capacity, and drives TLR4/MyD88-mediated inflammation (Table 1).

**Table 1:** Effects of AFB1 on intestinal barrier function and morphology in vivo and in vitro across different species.

Species/Model	AFB1 Dose	Experimental Period; Sample (N and Sex)	Intestinal Site/Model	Main Findings	References
Chicken	200 mg/kg	42 days; 240 (60/group) and male	Jejunum (mRNA expression)	↓ claudin-3, ↓ occludin, and ↑ claudin-2	[15]
Pigs (IPEC-J2, in vitro)	40 µg/kg	48 h; NR and N/A	Jejunal cell culture (mRNA expression)	↓ Bcl-2, ↓ zonula occludens-1, ↑ Bax, ↑ IL-6, and ↑ CASPASE-3	[33]
			Jejunal cell culture	↓ intestinal cell viability; ↑ necrotic cells, ↑ late apoptotic cells, and ↑ early apoptotic cells	
Pigs	280 µg/kg	102 days; 14 and male (barrows)	Jejunal mucosa (mRNA expression)	↓ zonula occludens-1	[34]
Chicken	250 µg/kg	19 days; 360 (60/group) and mixed	Jejunum	↓ villus height, ↓ villus width, ↓ VH:CD ratio, and ↑ crypt depth	[36]
Chicken	600 µg/kg	7 to 21 days; 156 and NR	Small intestine	↓ goblet cell count ↓ villus height, villus width, and VH:CD ratio; ↑ crypt depth; jejunum: ↓ villus height and VH:CD ratio; ↑ crypt depth and G2/M arrest, ↓ villus height, VH:CD ratio, and absorptive cell number; ↑ TUNEL-positive cells and apoptosis rate	[46] [45] [53]
Pigs (IPEC-J2, in vitro)	10 to 50 µg/kg	12 h; NR and N/A	Jejunal cell culture (mRNA expression)	↓ CASPASE-3, ↑ zonula occludens-1, and ↑ occludin at 10 µg/kg AFB1; ↓ mucin 2 at 20 µg/kg AFB1; ↑ Bcl-2 and ↑ Bax/Bcl-2 ratio at 30–50 µg/kg AFB1	[47]
Chicken	1200 ng AFB1/g	21 days; 100 (25/group) and male	Duodenum, jejunum, and ileum	shortened and fused microvilli, absent tight junctions, irregular denatured protein masses, and loss of epithelial polarity	[49]
Mice (C57BL/6J mice aged 6 weeks)	200 µg/kg	126 days; 8 and male	Intestine	impaired intestinal barrier; ↓ ZO-1 and occludin expression	[51]
Rabbits	25 µg/kg	42 days; 20 (AFB1 group; 120 total across 6 arms) and NR	Jejunum	↑ crypt depth, ↓ VH:CD ratio, ↓ sIgA and MUC2, ↑ serum DAO and D-lactate; ↓ ZO-1, occludin, and claudin-4	[52]
Chicken (Ross 708)	1500 µg/kg	20 days; 288 and male	Jejunum	↑ lactulose/rhamnose ratio	[54]
Chicken	60 to 120 µg/kg	21 days; 480 (48/group) and NR	Jejunum	↑ crypt depth at 60 µg/kg AFB1	[55]
			Ileum	↓ villus height at 120 µg/kg AFB1	
Chicken	42 µg/kg	42 days; 300 and mixed (half male, half female)	Jejunum	↓ villus height and VH:CD ratio; ↑crypt depth	[56]
Chicken	1000 µg/kg	35 days; 250 total and mixed (half male, half female)	Jejunum	↓ villus height, ↓ villus width, ↓ VH:CD ratio, and ↑ crypt depth	[57]
Chicken	394 to 1574 µg/kg	21 days; 336 (48/group) and NR	Jejunum	↓ villus height, ↓ VH:CD ratio, ↑ crypt depth, ↑ goblet cell count, and ↑ lamina propria lymphoid follicle diameter	[58]
Chicken	40 µg/kg	21 days; 480 (120/group) and female	Jejunum (mRNA expression)	↓ claudin-1, ↓ sIgA, and ↓ pIgR	[59]
Chicken	1000 µg/kg	42 days; 400 (50/group) and NR	Ileal mucosa (mRNA expression):	↓ occludin, ↓ claudin-1, and ↓ zonula occludens-1	[60]
Chicken	40 µg/kg	42 days; 200→160 and mixed (half male, half female)	Jejunum (mRNA expression)	↑ CASPASE-3	[61]
Pigs	180 µg/kg	48 days; N = 48 and mixed: (24 barrows, 24 gilts)	Jejunum	↓ villus height	[62]
Pigs	500 µg/kg	40 days; 72 (18/group) and male (uncastrated)	Small intestine	↓ fold size and ↑ villus height	[63]

NR = Not Reported; N/A = Not Applicable; ↓: Decreased Size; ↑: Increased Size.

## 3. Gut Microbiota Dysbiosis, Mucus Layer Disruption, and the Gut–Liver Axis

### 3.1. Mucus Dysbiosis and Altered Gut Microbiota Composition

Dysbiosis, characterized by disturbances in the composition and functional activity of the gut microbiota, has been increasingly implicated in a wide range of chronic conditions, including colorectal cancer, diabetes, neurodegenerative and psychiatric disorders, asthma, Crohn’s disease, irritable bowel syndrome, allergies, obesity, eczema, cardiovascular diseases, and hepatic encephalopathy [64,65,66]. It is characterized by a loss of beneficial commensals, the overgrowth of pathogenic species, or a reduction in overall microbial diversity [64].

In this context, AFB1 represents an important dietary contaminant that may contribute to the onset or aggravation of gut dysbiosis. Exposure to AFB1 has been shown to alter the composition of the gut microbiota and its metabolic activity, promoting the expansion of opportunistic and pathogenic microorganisms while weakening intestinal barrier function [67,68], although the affected bacterial groups vary according to the animal model and exposure conditions. In broilers, AFB1 increased the abundance of *Staphylococcus xylosus* and *Escherichia–Shigella* while reducing *Lactobacillus aviarius*. These changes were largely reversed by CMD supplementation. *L. aviarius* abundance was positively correlated with average daily gain, body fat, and SOD activity and negatively correlated with AFB1 residues and liver MDA, whereas *S. xylosus* and *Escherichia–Shigella* showed the opposite pattern. CMD also improved growth performance, reduced tissue damage, and helped maintain microbial stability, with 1 g/kg reported as the optimal dietary dose [56]. In turkey poults, AFB1 promoted dysbiosis and the growth of opportunistic bacteria, particularly *Streptococcus lutetiensis*. This increase may be linked to impaired nutrient absorption and immune function, which create favorable conditions for its proliferation [50]. Similarly, AFB1-treated rats showed a marked increase in *Alloprevotella* spp. and a microbial profile that differed from that of control animals. Since *Alloprevotella* produces succinic and acetic acids, its overgrowth may contribute to intestinal inflammation, tissue injury, and colitis, suggesting that dysbiosis is one pathway through which AFB1 damages the gastrointestinal tract [66]. Other studies have reported dose-dependent increases in Clostridiales and Bacteroidales, together with decreases in Lactobacillales, *Streptococcus* spp., and *Lactococcus* spp. However, some broiler studies found higher levels of Gram-negative bacteria and lactic acid bacteria at elevated AFB1 doses, along with increased volatile fatty acid production. AFB1 exposure has also been associated with a greater number of bacterial mutants, indicating that its genotoxic effects may extend to the gut microbiota itself [69].

This imbalance can trigger or aggravate intestinal inflammation, further compromising epithelial integrity. More broadly, the gut microbiome, including bacteria, viruses, fungi, protozoa, and parasites, plays an essential role in maintaining host homeostasis through complex symbiotic and pathogenic interactions [70,71]. Microbial metabolites derived from this ecosystem can influence distant organs and contribute to the development of systemic diseases [67,68,69,70].

Disruption of gut homeostasis has also been closely linked to liver pathology. Dysbiosis contributes to the progression of liver failure, and interventions aimed at restoring microbial balance have demonstrated beneficial effects in both experimental models and clinical settings [71,72]. Patients with liver failure or cirrhosis, as well as corresponding animal models, consistently exhibit compromised intestinal barrier function and evidence of bacterial translocation [73,74,75], although the precise characteristics of the translocated microbiota remain insufficiently defined.

### 3.2. Intestinal Permeability, Bacterial Translocation, and the Gut–Liver Axis

Along the same lines, AFB1 causes alterations in the integrity of the intestinal epithelial barrier by reducing the number of goblet cells, which results in a thinner protective mucus layer [46], and by decreasing the gene expression of tight junction proteins such as ZO-1, thereby weakening these intercellular connections and increasing intestinal permeability [33,34], commonly referred to as a “leaky gut,” which facilitates the passage of bacteria and microbial products beyond the intestinal lumen [37,38]. This process is closely linked to disruptions in tight junction proteins, which normally regulate paracellular transport and maintain barrier function [75]. Such impairment enables the movement of toxins and microorganisms into systemic circulation, contributing to bacterial translocation [76].

Evidence suggests that this increased permeability may also arise indirectly from AFB1 toxicity. Indeed, enhanced intestinal permeability has been associated with pathological processes affecting both the liver and kidneys [77]. Hernández-Ramírez et al.’s [32] experimental data further support this observation: broiler chickens exposed to AFB1-contaminated diets exhibited a marked increase in serum FITC-dextran levels to 2.4-fold higher than controls, which signifies important gastrointestinal leakage, whereas infection alone did not produce a comparable effect [32]. These results were similar to those reported by Nava-Ramírez et al. [50], both supporting the evidence that AFB1 alters the intestinal barrier, increasing its permeability, which facilitates the entry of luminal bacteria and their metabolites into the circulation, allowing dissemination to extraintestinal organs. Due to the anatomical connection via the enterohepatic circulation, the liver is particularly susceptible to microbial deposition [78]. This process is associated with elevated circulating levels of LPS and bacterial DNA, both of which contribute to inflammatory responses and disease progression [78]. While intrahepatic microbiota is thought to originate from circulating microbes following translocation, direct evidence supporting this mechanism in humans remains limited [78].

On another note, mucus dysbiosis associated with AFB1 exposure has also been observed in the colon. When the liver’s GSH pathway is overwhelmed by high doses of AFB1, it cannot eliminate all metabolites through the bloodstream; therefore, the excess conjugates are redirected into the bile for excretion [26,79,80]. The bile containing these conjugates is then secreted back into the intestinal tract and ultimately reaches the colon, where certain colonic bacteria producing enzymes such as β-glucuronidase can deconjugate these metabolites by removing the protective GSH or glucuronide groups [81]. This reaction releases the active toxic AFB1 molecule directly into the colonic lumen, creating a localized toxic environment. The reactivated AFB1 initially targets goblet cells and suppresses MUC2 expression, leading to thinning of the protective mucus layer that normally separates gut bacteria from the intestinal epithelium [3]. Once the mucus barrier is compromised, AFB1 induces oxidative stress through reactive oxygen species (ROS), which disrupts tight junction proteins such as ZO-1. This was suggested by Gao et al. [82], who showed that AFB1 decreases the expression of tight junction proteins, including claudin-1, claudin-3, claudin-4, and ZO-1, in CACO-2 cell lines.

As a result, intestinal permeability increases, leading to a “leaky colon” that allows pathogens and LPS to enter the bloodstream. This combination of epithelial barrier disruption and altered microbial composition may contribute to dysbiosis, with suppression of beneficial bacteria such as *Lactobacillus* and expansion of pathogenic species such as *Escherichia coli* and *Salmonella* [82,83].

### 3.3. Short-Chain Fatty Acids: A Mechanistic Link Between Gut Dysbiosis and the Gut–Liver Axis

Beyond the qualitative shifts in bacterial taxa described above, AFB1-induced dysbiosis also has a quantitative metabolic consequence that has received comparatively little attention in the AFB1 literature despite being central to gut–liver crosstalk: a reduction in microbially derived short-chain fatty acids (SCFAs), principally acetate, propionate, and butyrate. SCFAs are the main fermentation products of dietary fiber by saccharolytic gut bacteria and act on colonocytes and hepatocytes through G-protein-coupled receptors (FFAR2/FFAR3) and histone deacetylase inhibition; butyrate in particular supplies energy to colonocytes, upregulates MUC2 expression to reinforce the mucus layer, strengthens tight junction assembly, and exerts anti-inflammatory effects on mucosal and hepatic immune cells [84]. A reduction in SCFA-producing taxa therefore weakens the exact barrier and mucus mechanisms discussed in Section 3, Section 4.1 and Section 4.2, providing a plausible metabolic bridge between dysbiosis and barrier failure rather than two independent observations.

Direct evidence that AFB1 disrupts this pathway comes from a series of rodent studies. In male F344 rats, two weeks of oral AFB1 exposure reduced fecal SCFA levels by more than 70% in the high-dose group, together with the disruption of long-chain fatty acid and bile acid metabolism, in a manner that correlated inversely with AFB1 dose [85]. A related study by the same group showed that AFB1 induced inflammation-associated changes in the fecal lipidome, including a marked decrease in carbohydrate fermentation and SCFA output alongside a taxonomic shift toward a *Lachnospiraceae*/*Ruminococcaceae*-dominant, low-Lactobacillales profile resembling that seen in human inflammatory bowel disease [86], and dose-dependently reduced gut-microbial diversity while depleting lactic-acid-producing taxa [87]. These metabolic and taxonomic changes were paralleled, in mice and piglets, by disturbance of the gut microbiota–bile acid–farnesoid X receptor (FXR) axis, linking AFB1-induced dysbiosis mechanistically to hepatic bile-acid signaling and downstream liver injury [88,89].

**Figure 2:**
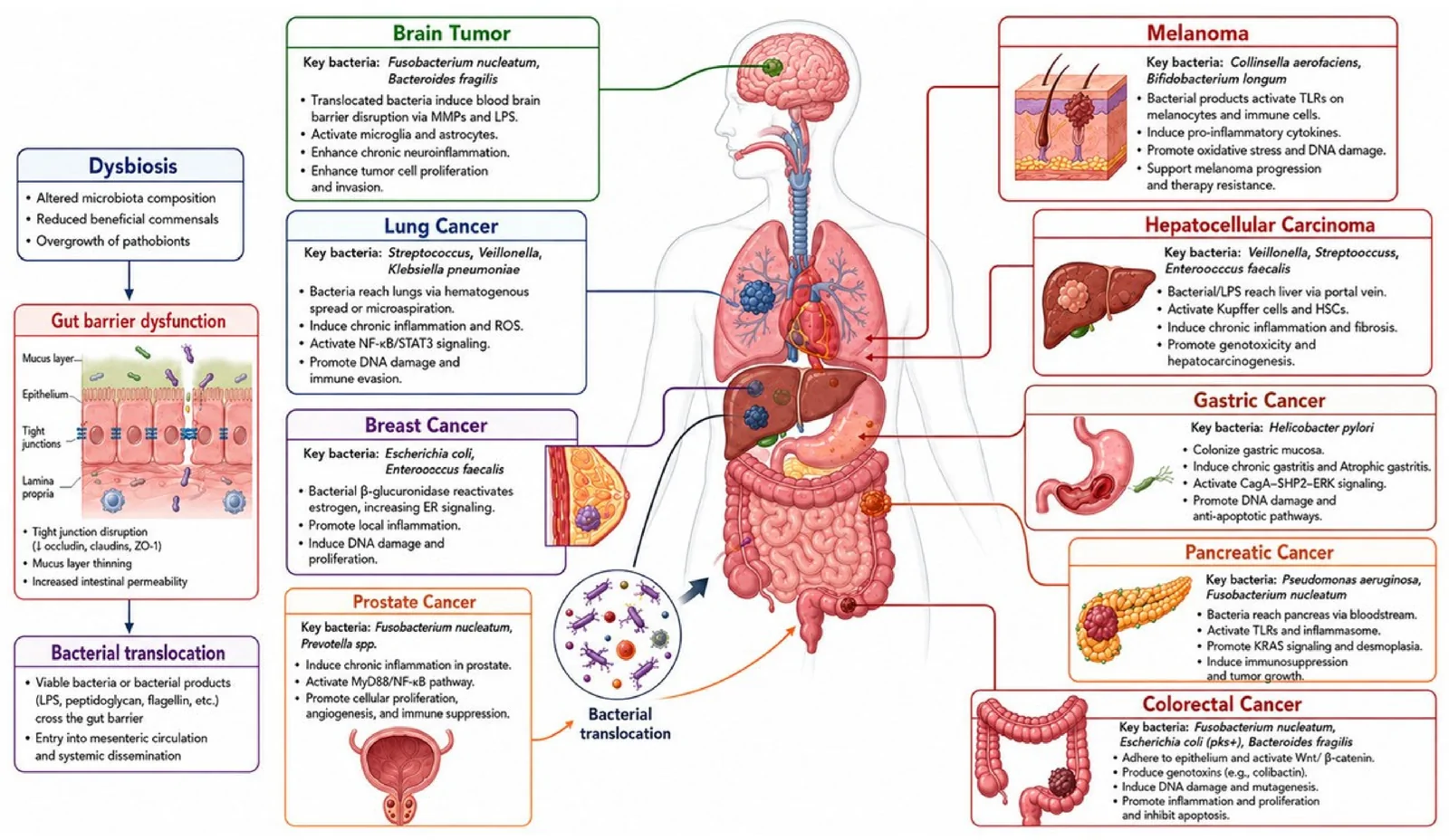
A summary of the different types of cancer caused by bacterial translocation from the gut microbiota.

Importantly, for the concern, raised by the reviewers, that this review remains largely descriptive and correlational, at least one study has moved beyond association to establish a causal role for the gut microbiota in AFB1-induced pathology using two complementary, interventional designs. Ye and colleagues showed that depleting the gut microbiota of mice with a broad-spectrum antibiotic cocktail attenuated AFB1-induced colonic barrier dysfunction and hepatic pyroptosis/inflammation, whereas fecal microbiota transplantation from AFB1-exposed donor mice reproduced colonic barrier dysfunction and liver injury in antibiotic-depleted recipients that had never themselves been exposed to AFB1 [25]. This antibiotic depletion/fecal transplantation design provides direct mechanistic, rather than purely correlative, evidence that AFB1-associated dysbiosis is sufficient to transmit barrier and inflammatory phenotypes and is, to our knowledge, the strongest available demonstration that the gut microbiota is not merely a bystander marker of AFB1 exposure but an active mediator of at least part of its toxicity. Because this evidence currently addresses hepatic rather than extraintestinal, non-hepatic carcinogenesis, we present it here as support for the plausibility of the gut–liver axis component of the proposed pathway (Figure 2) and flag, in Section 5.3, that equivalent depletion/transplantation experiments addressing extrahepatic tumor endpoints are still needed before the broader hypothesis linking AFB1, SCFA depletion, and cancers outside the liver can be considered more than plausible.

## 4. Tissue-Associated Microbiota and Extrahepatic Cancer Risk

The microbiota residing in our gastrointestinal (GI) tract and other sites may contribute to the prevention of tumorigenesis through several mechanisms, including the generation of beneficial oncometabolites such as butyrate, the maintenance of the integrity of the gut barrier, the modulation of immune cells to prevent inflammation through immune cells such as Treg cells, as well as the prevention of the colonization of the gut by pathogenic bacteria through competitive exclusion of pathogenic bacteria, thus inhibiting lethal infections by *Clostridioides difficile* [90]. Therefore, a balanced microbiome plays a crucial role in the maintenance of homeostasis [85,86]. Protective microbes like *Akkermansia*, *Ruminococcus*, and *Faecalibacterium* are related to promising immune inflection, meanwhile pro-tumor microbes like *Fusobacterium*, *Porphy-romonas*, *Helicobacter pylori*, and *Streptococcus* are associated with oncogenic inflammation, immune suppression, and tumor progression across multiple organ systems [91].

Notably, microbial signatures have also been identified in extraintestinal tissues and tumors. However, these observations do not establish whether microbial alterations precede tumor development, arise as a consequence of the tumor microenvironment, or represent associated changes [82]. In this context, several mechanisms have been proposed to explain how microbiota may influence carcinogenesis: low-grade inflammation caused by microbial antigens like LPS and flagellin, which activate the Toll-like receptors (TLRs) of the host cells [92]; the generation of potential oncometabolites such as hydrogen sulfide [90]; direct DNA-damaging effects of bacterial metabolites like colibactin and nitrosating compounds [93]; disruption of bile metabolism, leading to the production of carcinogenic secondary bile acids; compromised integrity of the GI barrier, which allows the transmigration of microbes and inflammatory mediators into the bloodstream [92]; and finally, the expression of virulence factors, such as the *E. coli* pks (polyketide synthase) pathogenicity island [90]. Indeed, pathogenic strains of *Escherichia coli* carrying the pks island are significantly more prevalent in the colonic mucosa of colorectal cancer patients (67%) compared to healthy individuals (21%) [94]. Collectively, these processes may contribute to a microenvironment that favors DNA damage, aberrant cell proliferation, and immune evasion [95].

Microbial genotoxins can form DNA adducts or induce DNA strand breaks, potentially resulting in mutations or chromosomal alterations when the damage is not adequately repaired, one of several mechanisms through which microorganisms may influence carcinogenesis. Genotoxins are destructive due to two main factors: they either form adducts or cause double-stranded breaks in DNA, which, when unsettled by normal DNA repair mechanisms, can present point mutations, insertions, deletions, or chromosomal rearrangements (inversions and translocations). Microbial genotoxins have the capacity to directly damage host DNA [96]. For instance, colibactin, produced by several *Enterobacteriaceae*, not just *E. coli* [97], induces double-strand breaks in host cells [82]. A similar ability to cause DNA damage has also been reported for the cytolethal distending toxin (CDT) produced by certain Proteobacteria [98].

Additionally, experimental models also suggest a link between microbiota-driven inflammation and tumor burden. For example, IL-10 knockout mice monoassociated with a mildly colitogenic strain of *Bacteroides vulgatus* develop an intermediate tumor load following AOM treatment. These findings suggest that the degree of microbiota-driven inflammation directly influences colorectal cancer susceptibility. The NF-κB pathway, central to innate immune signaling, serves as a key mechanistic bridge connecting microbiota-induced inflammation to colorectal tumorigenesis [99]. The chronic inflammation of IL-10 knockout mice apparently also increases pks oncogenesis.

On the other hand, *Enterococcus faecalis* is a commensal strain that makes large amounts of superoxide (O2−) at the luminal side of the colonic mucosa [90]. The fast O2− degradation can result in H_2_O_2_ that damages eukaryotic cellular DNA by forming point mutations, DNA–protein crosslinks, and DNA breaks. Similarly, the *Bacteroides fragilis* toxin (BFT) produced by enterotoxigenic *B. fragilis* (ETBF) enhances bacterial polyamine catabolism, generating ROS that damage host DNA and contribute to colon tumor formation [100]. ETBF also programs a pathogenic toxin that can induct TH17-mediated colitis, alongside colon-specific STAT3 activation and tumor formation in susceptible ApcMin mice, effects that can be reversed through IL-17 antibody blockade [101].

The gut microbiome has a dual aspect in oncology; first, it is seen as a role-player to oncogenesis, and second, as a contributing factor of oncotherapy results. In the context of AFB1 exposure, these mechanisms may be exacerbated by toxin-induced dysbiosis and barrier disruption, thereby amplifying carcinogenic processes beyond the liver. The following section will focus on specific cancers associated with microbiota alterations, with emphasis on mechanisms that may be linked to AFB1-induced dysbiosis.

### 4.1. Colorectal Cancer

The most well-known malignancy associated with microbiome dysbiosis is colorectal cancer (CRC). By binding E-cadherin via its FadA adhesin, triggering β-catenin signaling, and attracting tumor-associated myeloid cells that suppress antitumor immunity, *Fusobacterium nucleatum* promotes carcinogenesis [95]. Fragilysin, a metalloproteinase secreted by enterotoxigenic *Bacteroides fragilis*, increases permeability and inflammation by cleaving E-cadherin [102]. *Escherichia coli* that produces colibactin directly causes double-strand breaks in DNA, which fuels genomic instability [103,104]. Tumor-promoting inflammation is exacerbated when butyrate-producing taxa like *Faecalibacterium prausnitzii* are less abundant in CRC patients compared with healthy controls [105] because this reduces anti-inflammatory metabolites [95]. In addition, *Fusobacterium nucleatum* has also been related to the emergence of colorectal cancer (CRC) progression by binding to epithelial cells via its adhesin FadA, triggering Wnt/β-catenin signaling, and inhibiting natural killer (NK) cell activity [106]. Cumulatively, these mechanisms indicate that tissue-associated bacteria can induce colorectal carcinogenesis through direct genotoxicity, immune modulation, and barrier disruption, regardless of the bacteria’s original route of entry into the tissue.

### 4.2. Gastric Cancer

*Helicobacter pylori* infection is a well-known origin of gastric adenocarcinoma through chronic gastritis, inflammatory cytokine release, and DNA damage guided by virulence factors CagA and VacA [95]. With Cytotoxin-associated gene A (CagA) and Vacuolating cytotoxin A (VacA) virulence factors causing chronic gastritis, epithelial transformation, and DNA damage, *H. pylori* infection continues to be one of the most obvious examples of microbe-induced carcinogenesis [95]. In addition to *H. pylori*, dysbiosis with elevated *Prevotella* and nitrosating bacteria worsens mucosal damage and inflammation [107]. Because of these characteristics, gastric cancer is essential to understanding how gut microbial populations cause and maintain carcinogenesis [107]. The World Health Organization (WHO) has classified *H. pylori* as a class I carcinogen, making it the typical microbial carcinogen [108]. Its virulence components, CagA and VacA, cause DNA damage, trigger NF-κB and Mitogen-activated protein kinase (MAPK) signaling, and provide an environment that is pro-inflammatory in the stomach [95]. In addition to *H. pylori*, nitrosating bacteria (*Neisseria* and *Haemophilus*) that produce carcinogenic N-nitroso compounds are over-represented in stomach dysbiosis [107]. Moreover, *Streptococcus lutetiensis*, a bacterial species belonging to the human gut microbiota, has also been associated with gastric tumor progression through its ability to alter the immune response, thus promoting the development of tumors. Evidence suggests that *S. lutetiensis* provides tumor growth by disrupting infiltration and decreasing cytokine secretion of CD8^+^IL17A^+^ TRM cells within the gastric cancer microenvironment [109]. These findings demonstrate that both long-term colonizers (e.g., *H. pylori*) and transient tissue-associated species (e.g., *S. lutetiensis*) are capable of promoting gastric carcinogenesis by triggering chronic inflammatory and immune modulation.

### 4.3. Hepatocellular Carcinoma

The gut–liver axis illustrates the impact of microbial products and metabolites on hepatocellular carcinogenesis. Bile acid metabolism is altered by dysbiosis, which promotes carcinogenesis and impairs farnesoid X receptor/Takeda G protein-coupled receptor 5 (FXR/TGR5) signaling [110]. Significantly, the composition of the microbiome predicts the response to immunotherapy; positive results are linked to *Akkermansia muciniphila* and *Ruminococcaceae* [111]. Therefore, HCC is an example of how gut microorganisms affect immunological and metabolic interactions [95]. In HCC, modifications in bile acid metabolism facilitated by gut dysbiosis disorder the FXR-TGR5 signaling axis, promoting tumor growth [112]. This illustrates how microbial products, once they reach the liver, regardless of their source, can alter bile acid signaling and immune surveillance, thereby promoting tumor development.

### 4.4. Gallbladder Cancer (GBC)

GBC tissues contain significantly higher relative abundances of specific Actinomyces and *Streptococcus* species, including *S. anginosus*, *S. constellatus*, *S. intermedius*, *A. bowdenii*, *A. israelii*, and *A. gerencseriae,* compared with gallstone-associated chronic cholecystitis [113,114]. This malignancy was selected as an example illustrating how microbial alterations in the gut and biliary ecosystems can contribute to the development of a carcinogenic environment [95]. GBC has a substantial correlation with microbial changes linked to gallstone disease, although being little researched. Dysbiosis contributes to chronic cholecystitis and carcinogenesis by promoting the expansion of bile-resistant bacteria, such as *Enterobacter* and *Klebsiella*, while reducing the abundance of commensal microorganisms [95]. Bile microbes cause lithogenesis, change the metabolism of cholesterol, and produce secondary bile acids that can damage DNA [115]. Gallstone bacterial biofilms provide a carcinogenic niche by promoting chronic inflammation [116]. This example clarifies that bacterial colonization of a normally low-biomass biliary environment is acceptable to initiate a chronic inflammatory, carcinogenic niche.

### 4.5. Melanoma

Melanoma has offered some of the best evidence for the involvement of microbiomes in treatment, despite not being anatomically connected to the gut. Gut microbial diversity and the enrichment of taxa like *Collinsella aerofaciens*, *Bifidobacterium longum*, and *Akkermansia muciniphila* are strongly linked to the response to PD-1 inhibition. In one cohort of 42 metastatic melanoma patients, *C. aerofaciens* and *B. longum* were significantly more abundant among the 16 responders (38%) than the 26 non-responders (62%); *A. muciniphila* showed a consistent, though not independently significant, association with response, having been detected exclusively in responding patients [117,118]. Treatment sensitivity is restored by FMT from responders to non-responders [119]. The paradigm for immune system–microbiome interactions in oncotherapy that has been researched the most is melanoma [120]. This demonstrates that the presence and composition of tissue-associated microbiota can modulate tumor immune surveillance and susceptibility to therapy, regardless of the tumor’s tissue of origin.

### 4.6. Brain Tumor

Recent research indicates that the gut microbiome has a major impact on brain tumor development via the gut–brain axis, a bidirectional network that includes neuronal, immunological, and metabolic communication [121]. Dysbiosis changes systemic immunology, neuroinflammation, and the integrity of the blood–brain barrier (BBB), all of which are increasingly linked to the growth of gliomas and glioblastoma multiforme (GBM) [122]. Gut microorganisms create SCFAs such butyrate and propionate, which influence microglial activation and T-cell trafficking in the central nervous system (CNS) [123]. Loss of the SCFA-producing genus Faecalibacterium fosters a pro-inflammatory environment and is associated with tumor immune evasion in glioma [124]. Pathogenic bacteria and endotoxins can increase cytokine levels, including IL-6 and TNF-α, leading to increased glioma invasiveness [124]. This indicates that microbial pro-tumorigenic effect can extend even to anatomically shielded compartments such as the central nervous system, provided the relevant barriers are compromised.

### 4.7. Breast Cancer

Breast tissue and gut microbiota play critical roles in breast carcinogenesis by regulating estrogen metabolism, immunological function, and local inflammation [125]. Dysbiosis in the gut microbiome has been proposed to disturb the estrobolome, the collection of bacterial genes implicated in estrogen metabolism; however, a synthesis of nine case-control studies found substantial heterogeneity, with 71% of differentially abundant genera (45 out of 63) reported in only a single study and only *Escherichia coli* and *Roseburia inulinivorans* showing consistent, functionally relevant differential abundance between breast cancer cases and controls [118,126]. Increased β-glucuronidase activity promotes hormone-dependent tumor development by reabsorbing active estrogens [127]. *Methylobacterium radiotolerans* is more commonly found in breast cancer tissue than in normal tissue, detected in 100% of tumor samples, and the most significantly enriched taxon in tumor tissue (*p* = 0.0150) in one study [128], while Escherichia coli is similarly enriched in tumor tissue and has been shown to cause DNA double-strand breaks and oxidative stress [127]. Dysbiosis causes chronic inflammation and immunological dysregulation, which promotes tumor growth [129]. This illustrates that bacteria translocation to extraintestinal tissue can disrupt local hormone metabolism and create an inflammatory microenvironment, thereby promoting tumor initiation and progression.

### 4.8. Lung Cancer

The lung and gut microbiomes both play important roles in LC oncogenesis via inflammatory, metabolic, and immune-mediated mechanisms [130]. Dysbiosis is characterized by an increase in *Streptococcus*, *Veillonella*, *Prevotella*, *Enterobacteriaceae*, and *Bacteroides plebeius*, which promotes chronic airway inflammation and IL-17/IL-6-driven epithelial proliferation [130]. Reduced abundance of *Faecalibacterium* and *Roseburia* key butyrate producers reduces anti-inflammatory signaling, resulting in a tumor-friendly microenvironment [95]. Recent Mendelian randomization studies have found causative microbial taxa associated with LC risk. *Coprococcus*, *Holdemanella*, *Peptococcus*, and *Bacteroides clarus* were shown to be positively linked with LC susceptibility, whereas *Collinsella*, *Bifidobacteriaceae*, *Eubacteriaceae*, *Lachnospiraceae* UCG-010, and *Oscillibacter* were found to be protective [131]. This shows that microbial colonization of a distant organ system can maintain the chronic inflammatory signaling that drives oncogenesis.

### 4.9. Other Cancers

- Pancreatic Cancer

PDAC patients show distinct microbial fingerprints compared with healthy controls, including a significant increase in *Proteobacteria* (*p* = 0.013), specifically *Gammaproteobacteria* (*p* = 0.0062), significant enrichment of *Fusobacterium nucleatum* at the species level, and a significant decrease in short-chain fatty acid (SCFA) producers such as *Faecalibacterium* [132,133]. Microbial metabolites such TMAO and 3-IAA enhance tumor growth and disrupt the tumor immunological microenvironment [134]. The pancreatic tumor microbiome also reduces antitumor immunity by recruiting myeloid-derived suppressor cells (MDSCs) and activating TLRs [135]. This indicates that bacterial presence within pancreatic tissue drives a significant shift in the local immune microenvironment towards a pro-tumorigenic phenotype.

b. Prostate Cancer

Gut dysbiosis is associated with systemic inflammation and metabolic alterations that contribute to the development of prostate cancer [136]. Mycoplasma species have been linked to genomic instability and chronic inflammation of the prostate [96]. Emerging research suggests that microbiota may also influence testosterone metabolism and immunotherapy response, supporting its classification as a microbiome-associated cancer outside the GI tract [136]. This insinuates that extraintestinal bacterial colonization can promote carcinogenesis even in anatomically distant, non-contiguous organs.

Taken together, this evidence across ten organ systems establishes that upon translocating to extraintestinal sites, bacteria or their products possess a consistent capacity to promote oncogenesis via genotoxicity, immune modulation, and chronic inflammation, a mechanism (Figure 2) whose contribution and relevance to AFB1-induced pathology is explored next in Section 6.

## 5. Mechanistic Insights and Implications

### 5.1. AFB1’s Carcinogenic Potential Beyond HCC

Once ingested, AFB1 undergoes hepatic metabolism by CYP450 enzymes, resulting in the formation of aflatoxin B1-8,9-epoxide (AFBO), along with several hydroxylation products, including AFM1, AFQ1, and AFP1 [16,17]. Although AFB1 itself exhibits limited direct reactivity, its epoxide metabolite is highly reactive and forms adducts with DNA, RNA, and proteins, resulting in mutagenic, teratogenic, and carcinogenic effects [137].

Whilst AFB1 is well known for its hepatocarcinogenic effects, studies have shown that human exposure to AFs may also affect extrahepatic organs and contribute to other forms of cancer [138]. In fact, AFB1-DNA adducts can be identified in extrahepatic tissues including the colon, rectum, breast, and cervix, and significantly elevated levels in colorectal tumors have been found compared to adjacent normal tissue [139]. On another note, AFB1 also exerts multiple biological effects on the body, including the induction of inflammation, impairment of immune function, and disruption of natural physiological barriers such as the intestinal mucosal barrier. Its biological activity can directly or indirectly contribute to tissue damage.

For instance, AFB1 perturbs gut microbiota in sheep, causing inflammation and oxidative stress in the intestines [139], and demonstrates a significant association between inflammation and oxidative stress in rabbits [140].

AFB1 has also been shown to reduce beneficial microbes such as *Lactobacillus* spp. while promoting the proliferation of harmful bacteria, including *Escherichia coli*, in the small intestine of animals [61,83]. Its ability to compromise intestinal integrity is associated with a decrease in the relative gene expression of tight junction proteins such as ZO-1, which weakens these junctions and increases intestinal permeability [33,34]. These effects, summarized in Table 1, support a central role of AFB1 in disrupting intestinal barrier function.

AFB1 has also been reported to impair intestinal epithelial cell viability, increase apoptosis, and reduce goblet cell numbers, leading to thinning of the mucus layer [3,47]. Furthermore, experimental models have demonstrated that AFB1 exposure increases intestinal permeability and facilitates systemic dissemination of xenobiotics [141].

### 5.2. Indirect Cancer Risk

While aflatoxin B1 (AFB1) is a well-established hepatocarcinogen, primarily responsible for hepatocellular carcinoma development through metabolic activation and DNA adduct formation, emerging evidence suggests that its biological effects may extend beyond direct hepatic genotoxicity. However, the contribution of AFB1-induced extrahepatic mechanisms to cancer development remains incompletely understood and requires further investigation. Chronic AFB1 exposure has been shown to impair intestinal homeostasis by affecting epithelial integrity, immune responses, nutrient utilization, and microbial composition, potentially creating conditions associated with chronic inflammation and increased susceptibility to disease progression [76,82].

Experimental studies indicate that AFB1 exposure can compromise intestinal barrier function through alterations in tight junction proteins, induction of epithelial apoptosis, oxidative stress, and inflammatory responses [45,47,82]. These disturbances may increase intestinal permeability and contribute to microbial imbalance, which could influence host immune regulation and inflammatory pathways involved in carcinogenic processes [75,79,99]. Nevertheless, whether these intestinal alterations directly contribute to cancer initiation remains to be demonstrated.

The intestine represents a potential site of local AFB1 bioactivation because xenobiotic-metabolizing enzymes, including cytochrome P450 enzymes such as CYP3A4, are expressed in intestinal tissues [142]. Local formation of the reactive metabolite aflatoxin-8,9-epoxide (AFBO) could theoretically contribute to DNA damage within intestinal epithelial cells; however, the rapid turnover and continuous shedding of intestinal epithelial cells may limit the accumulation of transformed cells, which may partly explain the rarity of primary intestinal cancers compared with hepatocellular carcinoma [142]. Nevertheless, repeated exposure and persistent epithelial stress may contribute to long-term alterations in intestinal homeostasis.

At the molecular level, AFB1-derived reactive metabolites induce DNA adduct formation, oxidative stress, and genomic instability, which represent key mechanisms underlying its carcinogenic activity [17,19,23]. In addition, AFB1 exposure has been associated with altered regulation of apoptosis, cell cycle progression, and inflammatory signaling pathways, including pathways involving p53-mediated cellular responses [24,45,143]. These changes may contribute to a tissue environment favorable to carcinogenesis, although direct evidence linking intestinal AFB1 exposure to tumor development remains limited.

An additional potential mechanism involves AFB1-induced disruption of the intestinal barrier and microbiota composition, which may facilitate bacterial translocation and systemic inflammatory responses. Bacterial translocation and microbiota-derived factors have been increasingly implicated in cancer development through mechanisms involving immune activation, chronic inflammation, oxidative stress, and modulation of host signaling pathways [73,74,75,95]. Moreover, microbiota-derived metabolites and bacterial genotoxins have been associated with DNA damage and tumor-promoting effects in different tissues [81,94,99]. Therefore, AFB1-induced intestinal dysbiosis and barrier dysfunction could represent a potential indirect pathway linking toxin exposure with broader carcinogenic processes.

Overall, current evidence supports the concept that AFB1 may influence carcinogenesis through mechanisms extending beyond its classical hepatic effects; however, these extrahepatic pathways remain largely hypothetical and require validation in human populations. Future studies integrating toxicology, microbiome analysis, and longitudinal epidemiological approaches are needed to determine whether AFB1-induced intestinal alterations contribute meaningfully to cancer risk beyond hepatocellular carcinoma.

### 5.3. Strength and Limitations of the Proposed Evidence

The evidence supporting the proposed mechanisms varies in strength. Direct findings include restoration of barrier integrity by probiotic or adsorbent interventions (Table 1), reduced colitis-associated tumor development following IL-17 blockade in ETBF-colonized mice [101], and Th17-mediated inflammation after AFB1 exposure [52]. Colibactin-induced DNA damage [96,99] and BFT-related ROS production [100] are also supported experimentally, although mainly outside the AFB1 setting. By contrast, Treg-mediated immune suppression is inferred from non-AFB1 microbiome–cancer studies [99]. Most studies on AFB1-related dysbiosis remain correlational and cannot establish whether microbial changes cause or result from tissue injury.

Evidence for bacterial translocation is weaker because it relies largely on indirect markers, such as serum FITC-dextran [29,44] and circulating LPS or bacterial DNA [78], rather than direct detection of bacteria in tissues. Galarza-Seeber et al. [144] even reported no increase in permeability and reduced liver bacterial translocation in broilers exposed to 1–2 ppm AFB1. This suggests that translocation may depend on the dose, species, or experimental model.

The proposed pathway linking AFB1 exposure, barrier disruption, dysbiosis, bacterial translocation, and carcinogenesis should therefore be considered a working hypothesis. Barrier disruption and dysbiosis are the best-supported steps, whereas translocation remains uncertain, and its connection to extraintestinal carcinogenesis is mainly drawn from the broader microbiome–cancer literature. Direct studies confirming bacterial presence in extraintestinal tissues across different AFB1 doses are still needed.

A further limitation relates to the comparability of the evidence included in this review. The studies summarized in Table 1 involve several species, including chickens, turkeys, pigs, rabbits, mice, and rats, and use AFB1 doses ranging from ng/g to mg/kg, with exposure periods extending from a few hours in in vitro models to more than four months. Because poultry are considerably more sensitive to AFB1 than mammals, such wide differences in species, dose, and exposure duration make direct comparisons between studies difficult and limit the extent to which the findings can be extrapolated to humans. More importantly, all of the available evidence comes from animal studies or in vitro experiments. To date, no human studies have confirmed that AFB1 causes intestinal barrier disruption, dysbiosis, or bacterial translocation. The lack of human validation should therefore be considered a major limitation of the proposed model rather than a minor caveat. To formally evaluate the methodological quality of the animal studies summarized in Table 1, a risk-of-bias assessment was conducted using SYRCLE’s Risk of Bias tool for animal intervention studies (Table 2).

## 6. Conclusions

In conclusion, accumulating evidence indicates that aflatoxin B1 (AFB1) disrupts intestinal homeostasis by impairing epithelial barrier integrity, inducing oxidative stress and inflammation, and altering the gut microbiota across multiple experimental models. These findings suggest that the biological effects of AFB1 may extend beyond its well-established hepatotoxic and hepatocarcinogenic properties. Moreover, tissue-associated microbiota is increasingly recognized as a contributor to carcinogenesis in several organs through mechanisms involving chronic inflammation, immune modulation, and genotoxicity.

However, a major mechanistic gap remains. Although AFB1-induced intestinal barrier dysfunction and dysbiosis may favor bacterial translocation, direct evidence demonstrating that this process contributes to extrahepatic carcinogenesis in vivo is still lacking. Consequently, the proposed gut barrier–microbiota–carcinogenesis axis should be regarded as a biologically plausible hypothesis rather than an established mechanism. Future studies combining microbial tracing, multi-omics approaches, and advanced experimental models are needed to determine whether bacterial translocation represents a causal link between AFB1 exposure and systemic carcinogenesis.

While these mechanistic questions remain unresolved, minimizing dietary exposure to AFB1 through improved agricultural practices, appropriate storage conditions, and rigorous food safety monitoring remains the most effective strategy to reduce its adverse health effects. A better understanding of the interplay among AFB1 toxicity, intestinal barrier function, and the gut microbiota may not only clarify the systemic consequences of chronic AFB1 exposure but also identify novel biomarkers and microbiota-targeted interventions for cancer prevention.

## 7. Materials and Methods

### 7.1. Literature Search Strategy and Study Selection

The literature included in this comprehensive narrative review was identified through structured searches of PubMed/MEDLINE, Scopus, Web of Science, and Google Scholar, covering publications from January 2000 to July 2026. The search strategy combined the terms “aflatoxin B1” or “AFB1” with keywords related to intestinal barrier function, including “intestinal barrier”, “gut microbiota”, “dysbiosis”, “mucus”, “MUC2”, “tight junction”, “bacterial translocation”, “gut–liver axis”, “short-chain fatty acids”, “tissue microbiota”, “oncobiosis”, and “cancer”, using Boolean operators and appropriate combinations to maximize retrieval. To ensure comprehensive coverage, the reference lists of relevant original articles and recent reviews were also manually screened to identify additional eligible studies.

Original peer-reviewed articles, including in vivo animal studies, in vitro cell-based investigations, and available human studies, as well as high-quality review articles published in English, were considered for inclusion when they addressed the effects of AFB1 on intestinal barrier integrity, mucus layer function, gut microbiota composition or microbial metabolites, bacterial translocation, the gut–liver axis, or the relationship between tissue-associated microbiota and carcinogenesis. Studies involving other aflatoxins (e.g., AFM1) or other mycotoxins were included only when they provided relevant comparative information or methodological context and are explicitly identified as such throughout the manuscript. Conference abstracts, non-peer-reviewed preprints, and studies lacking accessible full-text articles were excluded.

Because much of the available in vitro evidence relies on the Caco-2 and IPEC-J2 intestinal epithelial models, the literature search was intentionally broadened to include studies employing additional experimental systems, such as HT-29, T84, NCM460, HCT116, SW480, IEC-6, and IPEC-1 cells. This approach allowed for a more comprehensive assessment of the strengths, limitations, and translational relevance of the current experimental evidence.

### 7.2. Data Analysis and Synthesis

Given the mechanistic scope of this review, the retrieved literature was analyzed qualitatively rather than quantitatively. Studies were critically evaluated according to their experimental model, AFB1 exposure conditions, biological endpoints, and principal findings. Particular attention was given to evidence describing alterations in intestinal barrier integrity, mucus production, tight junction proteins, oxidative stress, inflammatory responses, gut microbial composition and function, bacterial translocation, and the potential contribution of tissue-associated microbiota to carcinogenesis.

The available evidence was synthesized by integrating findings across cellular, animal, and human studies to identify areas of agreement, discrepancies among experimental models, and remaining knowledge gaps. When conflicting results were identified, differences in experimental design, exposure dose, treatment duration, biological model, and analytical methodology were considered during data interpretation. This integrative approach enabled the development of a comprehensive overview of current knowledge while highlighting limitations in the existing literature and priorities for future research.

## Figures and Tables

**Table 2:** Risk of bias assessment of studies included in Table 1, using SYRCLE’s Risk of Bias tool for animal studies.

Study (Ref.)	Species Model	Sequence Generation	Baseline Characteristics	Allocation Concealment	Random Housing	Blinding (Performance)	Random Outcome Assessment	Blinding (Detection)	Incomplete Outcome Data	Selective Reporting	Other Bias
[41]	Chicken (Cobb)	Unclear	Yes	Unclear	Unclear	Unclear	Unclear	Unclear	Yes	Yes	Ethics OK (Sichuan Agricultural University, Approval No. 2012-024); funded by Huimin Project of Chengdu Science and Technology (2016-HM01-00337-SF); no COI declared; no power justification
[40]	Chicken (Cobb)	Unclear	Yes	Unclear	Unclear	Unclear	Unclear	Unclear	Yes	Yes	Ethics OK (Sichuan Agricultural University); academic/government funding (Changjiang Scholars program, IRT 0848, Sichuan Province Education Dept); no COI declared; no power justification
[46]	Chicken (Cobb)	Unclear	Yes	Unclear	Unclear	Unclear	Yes	Unclear	Yes	Yes	Ethics OK (Sichuan Agricultural University, Approval No. 2012-024); academic/government funding (Changjiang Scholars, IRT 0848, Sichuan Province Education Dept, Chengdu Huimin project); no COI declared; no power justification
[54]	Chicken (Ross 708)	Unclear	Yes	Unclear	Unclear	Unclear	Unclear	Unclear	Yes	Yes	Ethics OK; mortality corrected; funding/COI not stated
[48]	Chicken	Unclear	Yes	Unclear	Unclear	Unclear	Unclear	Unclear	Yes	Yes	Ethics OK; no COI; funding not stated; later correction published
[49]	Chicken	Unclear	Yes	Unclear	Unclear	Unclear	No	Unclear	Unclear	Yes	Ethics OK; 2 co-authors industry-affiliated; mortality handling unclear
[50]	Chicken	Unclear	Yes	Unclear	Unclear	Unclear	No	Unclear	Unclear	Yes	Ethics OK; test products supplied by their own maker; no funding stated
[51]	Chicken	Unclear	Unclear	Unclear	Unclear	Unclear	Unclear	Unclear	Unclear	Yes	Incomplete excerpt reviewed; ethics OK; most domains unclear from this gap
[36]	Chicken	Unclear	Yes	Unclear	Unclear	Unclear	Yes	Unclear	Yes	Yes	Ethics OK (King Saud University); govt funded (MAARIFAH); no COI declared; unit of analysis explicitly addressed (pen for performance, animal for other measures); no power justification
[52]	Chicken	Unclear	Yes	Unclear	Unclear	Unclear	Yes	Unclear	Yes	Yes	From a search snippet, not the full PDF; ethics/funding not stated
[53]	Chicken	Unclear	Yes	Unclear	Unclear	Unclear	Unclear	Unclear	Yes	Yes	Ethics OK; no COI; academic funding; some assays used subsamples
[15]	Chicken	Unclear	Yes	Unclear	Unclear	Unclear	Unclear	Unclear	Yes	Yes	Ethics OK; 2 co-authors from binder maker (Trouw Nutrition) despite paper declaring no conflicts of interest; funding source not stated
[55]	Chicken	Unclear	Yes	Unclear	Unclear	Unclear	Unclear	Unclear	Yes	Yes	Ethics OK (Henan Agricultural University); academic/government funding (Henan Province NSF); no COI declared; two-stage design used separate cohorts; no power justification
[44]	Chicken	Unclear	Yes	Unclear	Unclear	Unclear	Yes	Yes	Unclear	Yes	Ethics OK; double-blind histopathology eval; funding not stated
[46]	Mice (C57BL/6J)	Unclear	Yes	Unclear	Unclear	Unclear	Unclear	Unclear	Unclear	Yes	No study-specific ethics # shown; funding not stated; no attrition data
[47]	Rabbits	Unclear	Yes	Unclear	Unclear	Unclear	Unclear	Unclear	Unclear	Yes	Ethics OK; funding/COI not stated
[34]	Pigs	Unclear	Yes	Unclear	Unclear	Unclear	Yes	Unclear	Yes	Yes	Ethics OK; govt funded
[39]	Pigs (IPEC-J2, in vitro)	Unclear	Yes	Unclear	Unclear	Unclear	Yes	Unclear	Yes	Yes	Combined in vitro/mouse study; mouse arm judged; ethics OK
[33]	Pigs (IPEC-J2, in vitro)	Unclear	Yes	Unclear	Unclear	Unclear	Yes	Unclear	Yes	Yes	In vitro study—housing/blinding domains N/A; industry-affiliated co-authors
[56]	Pigs	Unclear	Yes	Unclear	Unclear	Unclear	Yes	Unclear	Yes	Yes	Industry co-funded (Alltech); one co-author is an Alltech employee
[57]	Pigs	Unclear	Yes	Unclear	Unclear	No	Unclear	No	Yes	Yes	No funding declared; no power justif.

Yes = low risk of bias; No = high risk of bias; Unclear = insufficient information reported to permit judgement. Domains follow SYRCLE’s Risk of Bias tool for animal intervention studies [145]. “Other bias” details are summarized within each row’s cell. **COI** = conflict of interest; **IRT** = Innovative Research Team; **NSF** = Natural Science Foundation.

## Data Availability

No new data were created or analyzed in this study. Data sharing is not applicable to this article.
